# ﻿Five alien achatinid land snails (Gastropoda, Eupulmonata) first reported in greenhouses of Italian botanical gardens

**DOI:** 10.3897/zookeys.1208.119147

**Published:** 2024-07-29

**Authors:** Giuseppe Manganelli, Andrea Benocci, Debora Barbato, Folco Giusti

**Affiliations:** 1 Dipartimento di Scienze Fisiche, della Terra e dell’Ambiente, Università di Siena, Via Mattioli 4, 53100 Siena, Italy Università di Siena Siena Italy; 2 NBFC (National Biodiversity Future Center), Palermo, Italy NBFC (National Biodiversity Future Center) Palermo Italy; 3 Museo di Storia Naturale dell’Accademia dei Fisiocritici, Piazzetta S. Gigli 2, 53100 Siena, Italy Museo di Storia Naturale dell’Accademia dei Fisiocritici Siena Italy

**Keywords:** Geographical distribution, non-indigenous molluscs, shell and genital morphology, taxonomy, translocated species

## Abstract

Plant trade and exchange for horticulture, recreation or research play a significant role in the dispersal of molluscs. Alien slugs and snails accidentally introduced into Europe have established rich communities in several countries, but although these introductions could have ecological and economic implications, mollusc xenodiversity in Italian botanical gardens, plant nurseries, and greenhouses has never been investigated. Facilities throughout the country were therefore visited between 2017 and 2023. Here the list of the achatinoidean species so far recorded from Italian greenhouses is provided, giving a short description of their diagnostic characters. The greenhouses of Trento and Padua host interesting assemblages of achatinoideans: the results of this study include the first reports of four achatinids, namely *Allopeasclavulinum*, *Opeashannense*, *Paropeasachatinaceum* and *Subulinaoctona* from Italy and the first record of *Geostilbiaaperta* from Europe. Reproductive anatomy of all species except *Geostilbiaaperta* is illustrated in detail, integrating anatomical knowledge of this group of tiny molluscs, some of which are known mainly from their shell characters. The systematics of all the species is discussed, highlighting topics for future research (e.g., status of *Allopeasmauritianum*, real identity of *Helixhannense*, anatomy of *Opeashannense*, species-level taxonomy of *Subulina*, systematic relationships and species-level taxonomy of *Geostilbia*).

## ﻿Introduction

An increasing number of organisms of limited mobility are rapidly spreading outside their native range and habitat (Bergey et al. 2014; Pergl et al. 2017; Hulme 2021) due to the global expansion of trade and transport of goods (Hulme 2009; Seebens et al. 2017). This may have dramatic implications for local biodiversity, food crops, human health, ecosystem processes and habitat degradation (Kenis et al. 2009; Keller et al. 2011). In temperate countries, a major component of xenodiversity consists of tropical species unwittingly introduced with plants and substrates for horticulture or recreation. Botanical gardens and hothouses play a major role in the accidental introduction of foreign species (e.g., Teodorescu and Matei 2010; Heywood 2011; Szczepkowski et al. 2014; Hulme 2015; Kolicka et al. 2015; Wang et al. 2015; Jaskuła et al. 2019).

Alien slugs and snails have already established rich communities in European plant nurseries, greenhouses, and botanical gardens (e.g., Da Sois 2015; Reischütz et al. 2018; Kwitt et al. 2019) and new species arrive every year (e.g., von Proschwitz 2004; Preece and Naggs 2014; Jaskuła et al. 2019; Richling and von Proschwitz 2021). Land molluscs are easily transported with living plants via eggs in root balls or hatched individuals on leaves, stems, moss on pots, decaying plant matter on the soil surface or in the substrate (Robinson 1999; Bergey et al. 2014). However, despite the important ecological and economic implications of the introduction of alien gastropods, the non-native mollusc communities of Italian botanical gardens have not been thoroughly investigated. Therefore, our research group tried to fill this gap, visiting facilities throughout the country: here we report the rich assemblage of achatinoideans found in the first years of this study, including the first European record of *Geostilbiaaperta* (Swainson, 1840).

Achatinoideans include several travelling snails (e.g., species that have the ecological flexibility to survive long journeys and spread and prosper in their new habitats; Smith 1989) that have been unintentionally introduced outside their native range through plant trade and exchanges: some have now achieved a pantropical distribution, while others, widespread in greenhouses and plant nurseries, have become cosmopolitan and occur in very high densities in such anthropogenic habitats. So far seven species have been recorded from European greenhouses and hothouses: *Allopeasclavulinum* (Potiez & Michaud, 1838), *Allopeasgracile* (Hutton, 1834), *Leptinariaunilamellata* (d’Orbigny, 1838), *Opeashannense* (Rang, 1831), *Paropeasachatinaceum* (Pfeiffer, 1846), *Subulinaoctona* (Bruguière, 1789), *Subulinastriatella* (Rang, 1831) (Horsák et al. 2020).

As pointed out by Horsák et al. (2020) and Richling and von Proschwitz (2021), it is not easy to distinguish these small similar-looking snails, especially for scientists in countries where such genera and species are not native. Tiny achatinoideans include many described taxa, the great majority of which live in tropical areas relatively neglected by research biologists. The taxonomy of this speciose group is still being defined and shell-based identifications are not considered very reliable (Solem 1989; Naggs 1994; von Proschwitz 1994; Cowie 1997; Thompson 2011; Brodie and Barker 2012; Gittenberger and van Bruggen 2013; Budha et al. 2015; Horsák et al. 2020; Richling and von Proschwitz 2021). There is evidence that a conchological approach has often caused generic misallocations and species misidentifications (Cowie 1997, 2000; Gittenberger and van Bruggen 2013; Budha et al. 2015).

Anatomical dissection of small achatinoideans is not easy: the proximal part of the penial complex is difficult to interpret since its components are so reduced and closely packed as to appear a single structure. Nor are anatomical differences between extant genera very evident. Nevertheless, although anatomical study may only provide partial solutions, it is absolutely necessary because generic taxa were defined on anatomical characters so that identification is only possible by this examination. The aim of the paper is to list the species of achatinoideans so far recorded from Italy, giving a short description of their diagnostic shell and anatomical characters in order to facilitate identification, and understanding of their taxonomic and nomenclatural framework.

## ﻿Materials and methods

Between 2017 and 2023, the tropical greenhouses of seven facilities in central and northern Italy (Science Museum of Trento (MUSE); botanical gardens of Siena, Florence, Pisa, Padua, Bologna, and Milan) were visited and inspected for alien molluscs as part of the XenoDOMuS project on xenodiversity in tropical greenhouses of Italian botanical gardens and scientific museums.

Snails and slugs were detected by visual search and by collecting leaf litter and surface soil. The visual search enabled detection of larger specimens, while collection of litter and soil revealed smaller species. The litter and soil were dried and sieved through decreasing mesh sizes. The coarser fraction was sorted visually, the others under a stereomicroscope. Although this method involves a huge investment in terms of sampling effort and time, it ensures more efficient collection of small mollusc species than a visual search.

Identifications were based on morphological features (i.e., shell and anatomical characters). Live specimens were drowned in water, then fixed and preserved in 75% ethanol buffered with sodium carbonate. The bodies were dissected under a light microscope using fine-pointed watchmaker’s forceps. Anatomical organs were drawn using a camera lucida. Anatomical nomenclature usually followed the standard references for Eupulmonata (e.g., Manganelli et al. 2022 and references therein); as regards the proximal penial complex, the terms epiphallus and penial caecum are in customary use in the “subulinids” (e.g., Naggs 1994; Gittenberger and van Bruggen 2013).

The descriptive terms (e.g., short vs long, slender vs wide) refer to comparisons of the same section of the genitalia in different taxa of achatinids. The directional terms proximal, basal, and initial denote the part closer to the gonad, whereas distal, apical, final, and terminal denote the part closer to the gonopore in the case of ducts of the main axis of the genitalia (e.g., free oviduct, vagina, vas deferens, penis). The same terms denote the part closer to (proximal, basal, or initial) or further from (distal, apical, final, or terminal) the main axis of the genitalia in the case of blind structures radiating from the main axis of the genitalia (bursa copulatrix, penial/vaginal/atrial appendix, etc.).

The material is stored in the G. Manganelli collection (GMC; Dipartimento di Scienze Fisiche, della Terra e dell’Ambiente, Università di Siena, Italy).

Acronyms of the shell variables:

**AH** aperture height,

**AW** aperture width,

**SD** shell diameter,

**SH** shell height.

Acronyms of the anatomical organs:

**AG** albumen gland,

**BC** bursa copulatrix (gametolytic gland),

**BW** body wall,

**DBC** duct of bursa copulatrix,

**DP** distal penis,

**DV** distal vagina,

**Eg** egg/eggs in the uterine spermoviduct,

**Ep** epiphallus,

**FHD** first hermaphrodite duct,

**FO** free oviduct,

**GA** genital atrium,

**LB** vaginal lateral bulge,

**P** penis,

**PC** penial caecum,

**PP** proximal penis,

**PR** penial retractor,

**PS** penial sheath,

**PSO** prostatic spermoviduct,

**SOD** spermoviduct (ovispermiduct; second hermaphrodite duct),

**USO** uterine spermoviduct,

**V** vagina,

**VD** vas deferens.

## ﻿Results

Non-native molluscs were observed in all the facilities investigated, but achatinids were only found in Padua (3 species: *Allopeasclavulinum*, *Paropeasachatinaceum*, *Subulinaoctona*) and Trento (4 species: *Allopeasclavulinum*, *Opeashannense*, *Subulinaoctona*, *Geostilbiaaperta*). In these two greenhouses, high densities of small achatinids and other exotic slugs, snails, and soil invertebrates were found. The systematics, morphology, ecology, and distribution of the five species of achatinids are described below. A small undescribed slug of the little-known systellommatophoran family Rathouisiidae found in the tropical greenhouse of the Science Museum of Trento (MUSE) has already been described (Manganelli et al. 2022).

### ﻿Family Achatinidae Swainson, 1840

The superfamily Achatinoidea Swainson, 1840 has a complex and still unresolved systematic framework. Nordsieck (1986) included five families in this group (Achatinidae Swainson, 1840, Ferussaciidae Bourguignat, 1883, Subulinidae Fischer & Crosse, 1877, Coeliaxidae Pilsbry, 1907, and Thyrophorellidae Girard, 1895), while Bouchet and Rocroi (2005) listed only four (Achatinidae, Ferussaciidae, Subulinidae, Micractaeonidae Schileyko, 1999). On the other hand, Schileyko (1999) only included the Achatinidae in the Achatinoidea, and grouped Subulinidae, Glessulidae Godwin-Austen, 1920, Micractaeonidae, and Ferussaciidae in their own superfamily, Subulinoidea Fischer & Crosse, 1877. Fontanilla et al. (2017) published a multi-gene phylogenetic analysis of the achatinoideans examining 24 taxa from five achatinoid families (Achatinidae, Coeliaxidae, Ferussaciidae, Subulinidae, and Thyrophorellidae). The results suggest that the family level systematics requires a radical re-evaluation because among the traditionally recognised families, only Achatinidae are monophyletic whereas Ferussaciidae, Coeliaxidae, and Subulinidae are polyphyletic or unresolved and members of the Coeliaxidae and Thyrophorellidae cluster among the subulinids.

In their “Revised classification, nomenclator and typification of gastropod and monoplacophoran families”, Bouchet et al. (2017), following Fontanilla et al. (2017), moved the Subulinidae, Coeliaxidae, Thyrophorellidae, and the ferussaciid genus *Cecilioides* Férussac, 1814 to the Achatinidae. However, in the absence of a more comprehensive molecular study of members of all the families and subfamilies previously recognised in the achatinoideans, they proposed a preliminary classification in which most of the subfamilies listed by Schileyko (1999) were treated as valid and allocated *Cecilioides* to a distinct subfamily (Cecilioidinae Mörch, 1864). It is nonetheless possible that the removal of *Cecilioides* from the Ferussaciidae was based on a misidentification of the species examined by Fontanilla et al. (2017) (E. Neubert, pers. comm. 7 May 2024). Indeed MolluscaBase (MolluscaBase eds 2024a) still lists *Cecilioides* in the family Ferussaciidae, albeit citing Bouchet et al. (2017) as the source for the page dealing with Achatinidae. Thus, in the absence of a more consistent classification at subfamily level, the species considered in the present study are simply taken in alphabetical rather than systematic order, with the exception of *Geostilbia* Crosse, 1867, traditionally considered a ferussaciid based solely on shell characters, which we consider last. The inclusion of *Geostilbia* in the ferussaciids is based on its close shell similarity with *Cecilioides*, but without anatomical and molecular evidence, its attribution to a different group/superfamily cannot be excluded.

#### ﻿ Allopeas clavulinum

Taxon classificationAnimaliaStylommatophoraSubulinidae

(Potiez & Michaud, 1838)

AC961840-E0DA-51D1-9C3B-7DCD7D15F454

Bulimus clavulinus Potiez & Michaud, 1838, 1: 136, pl. 14, figs 9, 10. Type locality: “L’île Bourbon”, namely Réunion Island, Mascarene Archipelago. Type material: lost (Smith 1992: 309).

##### Material examined.

Italy • 6 shells and 6 spirit specimens; Trento, Tropical greenhouse of the Science Museum of Trento (MUSE); 46°03'45.16"N, 11°06'50.08"E; 14 Dec. 2017; A. Benocci, G. Manganelli, D. Miserocchi leg.; GMC 47556 • 623 shells, 10 spirit specimens; same locality; 04 Jan. 2019, 10 Feb. 2019, 04 May 2019; D. Barbato, G. Bolzonella leg.; GMC 51194 • 418 shells, 2 spirit specimens; same locality; 01 Feb. 2022; D. Barbato, A. Benocci leg.; GMC 51184 • 133 shells; same locality; 02 Feb. 2023; F. Rossi leg.; GMC 57343 • 155 shells; same locality; 9 Feb. 2023; D. Barbato, A. Benocci leg.; GMC 57350 • 145 shells and 1 spirit specimen; Padua, Biodiversity Garden (Botanical Garden of Padua); 45°23'52.59"N, 11°52'50.37"E; 06 Mar. 2019; D. Barbato leg.; GMC 57373.

##### Description.

**Figures 1–8.**
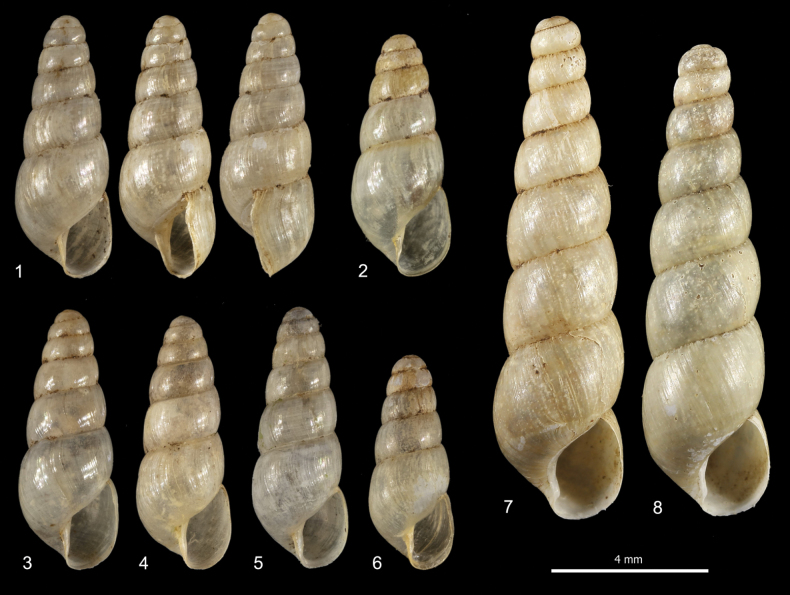
Shells of alien achatinids in Italian greenhouses: **1–6** shells of *Allopeasclavulinum* from the tropical greenhouse of the Science Museum of Trento (MUSE), D. Barbato & G. Bolzonella leg. 04 May 2019 **7, 8** shells of *Subulinaoctona* from the tropical greenhouse of the Science Museum of Trento (MUSE), D. Barbato & G. Bolzonella leg. 04 May 2019.

***Shell*** (Figs 1–6). Dextral, small, minutely perforate to imperforate, elongate, slender, conical, rather robust, pearly off-white, glossy and sub-transparent when fresh, with 5–7 slightly convex whorls, separated by moderately deep sutures. Apex obtuse, rounded, and smooth. Last whorl ~ 1/2 of shell height. Aperture small, ~ 1/3 of shell height, obliquely pyriform, slightly prosocline. Peristome interrupted, not thickened, only slightly reflected on columella, sometimes with slightly evident callous rim on parietum; columella straight; outer margin sinuous in lateral view (approximately inverse S-shaped). Protoconch smooth; teleoconch with thin and irregular collabral growth lines. Shell dimensions: SH 5.5–6.8 mm; SD 2.2–2.6 mm; AH 1.9–2.3 mm; AW 1.2–1.4 mm.

**Figures 9–11.**
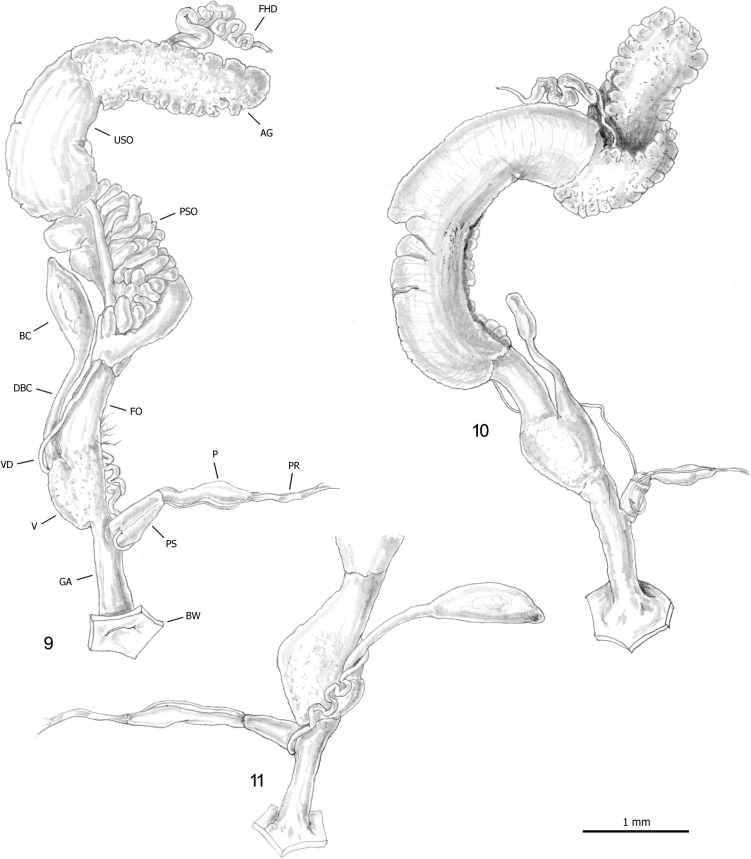
Genital anatomy of *Allopeasclavulinum* from the tropical greenhouse of the Science Museum of Trento (MUSE), D. Barbato & G. Bolzonella leg. 04 May 2019: **9, 10** genitalia (hermaphrodite gonad excluded) **11** distal genitalia.

**Figures 12–14.**
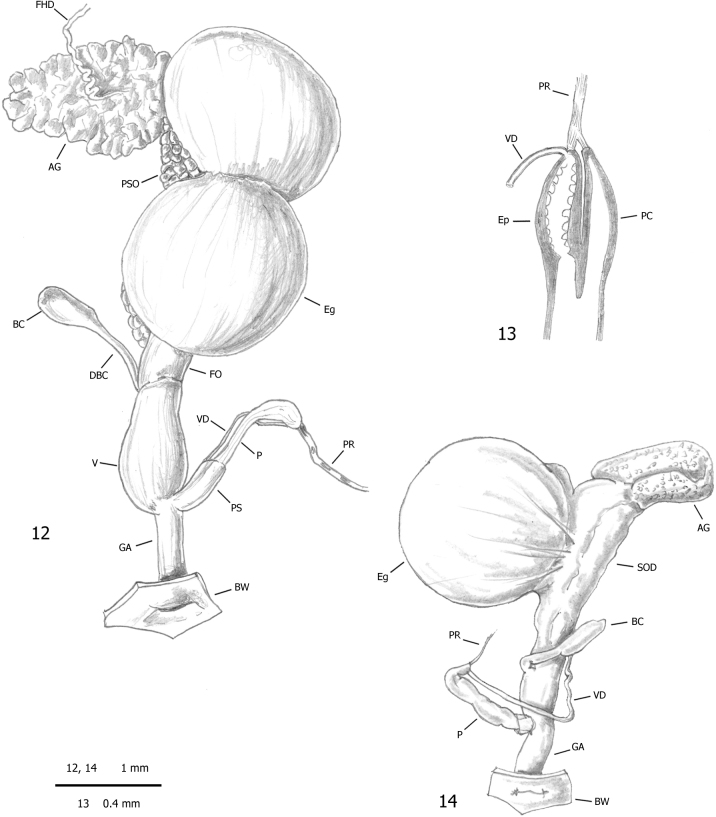
Genital anatomy of alien achatinids in Italian greenhouses: **12, 13** genitalia (hermaphrodite gonad excluded) and diagram of the proximal penial complex of *Allopeasclavulinum* from the tropical greenhouse of the Science Museum of Trento (MUSE), D. Barbato & G. Bolzonella leg. 04 May 2019 **14** genitalia (hermaphrodite gonad excluded) of *Opeashannense* from the tropical greenhouse of the Science Museum of Trento (MUSE), D. Barbato & G. Bolzonella leg. 04 May 2019.

***Female distal genitalia*** (Figs 9–12). Free oviduct long and wide. Bursa copulatrix sac-like, oval with long slender duct (slightly longer than bursa copulatrix), sometimes initially flared. Vagina short and wide (approximately as long as free oviduct) with small lateral bulge.

**Figures 15, 16.**
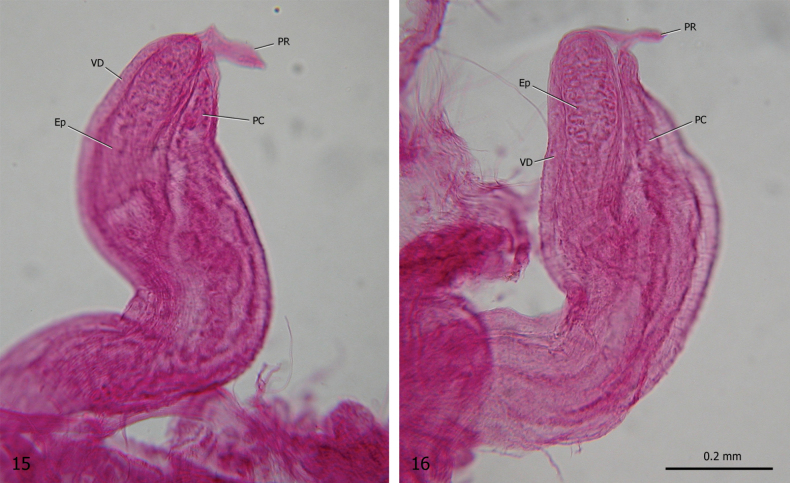
Proximal penial complex of *Allopeasclavulinum* from the tropical greenhouse of the Science Museum of Trento (MUSE), D. Barbato & G. Bolzonella leg. 04 May 2019.

***Male distal genitalia*** (Figs 9–13, 15, 16). Vas deferens almost uniform in diameter (very thin to thin along its entire length), entering penial complex at its proximal end. Penial complex consisting of epiphallus, penial caecum and penis. Epiphallus very short. Penial caecum very short (as long as epiphallus). Penis short to rather long, almost uniformly cylindrical, undivided, with penial sheath enveloping its distal tract. Penial retractor muscle bifid, one branch inserted on proximal end of epiphallus, one branch on tip of penial caecum.

***Genital atrium*** (Figs 9–12). Rather long.

##### Remarks.

Although *Allopeasclavulinum* is a well-known greenhouse snail (Kerney and Cameron 1979), its taxonomic and systematic placement is still not definitive, nor is its native range clear. The species was first described from Réunion Island (as Île Bourbon), Mascarene archipelago, in the western Indian Ocean (Potiez and Michaud 1838), but Griffiths and Florens (2006) thought it an East African species introduced into the Mascarene islands. The hypothesis that the species is native to Africa or East Africa was also maintained by Kerney and Cameron (1979), Robinson (1999), Probst (2001), Shea (2007), Cowie et al. (2008), Stanisic et al. (2010), and Foon et al. (2017). However Rowson et al. (2010) observed that Verdcourt, familiar with *A.clavulinum* in botanic gardens of the UK, never reported it from East Africa (e.g., Verdcourt 1983, 2000, 2006). Alternatively an Asian / south-east Asian origin was proposed by Brook et al. (2010) and Rumi et al. (2010). Support for a south-east Asian origin could come from the putative finding of a shell in the Holocene of Thailand (Robba et al. 2007) and its membership of a molecularly based monophyletic group, including other species from Sri Lanka (Fontanilla et al. 2017).

Today, *Allopeasclavulinum* occurs in humid tropical and subtropical lowlands across the world. It is reported from the West Indies, South America, West Indian Ocean islands, South and South-east Asia, New Guinea, Australia, and Pacific islands. Outside the tropics and subtropics it only occurs in heated greenhouses or very disturbed habitats, mainly in the northern hemisphere. Indeed it has been reported from North America, Europe, the Middle East, and New Zealand (see Table 1 for details and references). An alleged distinct subspecies *Allopeasclavulinumkyotoense* (Pilsbry & Hirase, 1904) is reported from Korea and Japan (Minato 1988; Noseworthy et al. 2007).

**Table 1.** Geographical distribution of *Allopeasclavulinum*. Asterisks indicate countries / islands where the species has been recorded only in greenhouses or very disturbed anthropogenic habitats.

Regions	Countries / Islands	References
**North America**	United States*	Dundee (1974), Nekola (2014)
**West Indies**	Hispaniola	Espinosa and Robinson (2021)
**South America**	Brazil and Suriname	Marcus and Marcus (1968), van Regteren Altena (1975), Simone (2006), Rumi et al. (2010)
**Europe**	Austria*, Czech Republic*, Finland*, Germany*, Great Britain*, Ireland*, Italy*, Netherlands* and Sweden*	Kerney and Cameron (1979), von Proschwitz (1994), Leiss and Reischütz (1996), Horsák et al. (2004, 2020), Da Sois (2015), Reischütz et al. (2018), Anderson and Rowson (2020), this paper
**Indian Ocean**	Madagascar, Mascarene, and Seychelles	Probst (2001), Gerlach (2006), Griffiths and Florens (2006), Bank and Menkhorst (2008), Emberton et al. (2010)
**West Asia**	Israel*	Mienis et al. (2012)
**South Asia**	Nepal	Budha et al. (2015)
**South-East Asia**	Indonesia, Peninsular Malaysia, Philippines, Sabah in Malaysian Borneo and Singapore	Ho (1995), Foon et al. (2017), Phung et al. (2017), Nurinsiyaha and Hausdorf (2019), Parcon et al. (2020)
**Oceania**	Australia, New Guinea, New Zealand* and Pacific islands (American Samoa, Chilean Islands: Rapa Nui, Cook Islands, Fiji, French Polynesia, Hawaii, Norfolk Island, Pitcairn Islands and Tonga)	Anonymous undated, van Benthem Jutting (1964), Solem (1989), Cowie (2000, 2001), Shea (2007), Stanisic et al. (2007, 2010), Brook et al. (2010), Brook (2014), Cowie et al. (2017), Maynard et al. (2018), Osorio (2018)

The genital anatomy of *Allopeas* species is poorly understood. Earlier studies have been performed on specimens of *Allopeasgracile* (Hutton, 1834) from Puerto Rico (Baker 1945; Baker in Pilsbry 1946) and Suriname (Gittenberger and van Bruggen 2013), *Allopeasclavulinum* from Scotland (Baker 1945; Baker in Pilsbry 1946) and Brazil (Marcus and Marcus 1968), *Allopeasmauritianum* (Pfeiffer, 1853) from Mauritius (Baker 1945; Baker in Pilsbry 1946) and *Allopeas* “spec. 2 and *Allopeas* spec. 3” from Pemba (Gittenberger and van Bruggen 2013). The overall distal genitalia organisation of our specimens (Figs 9–12) is consistent with that described in *Allopeasgracile* (Baker 1945: 88–89; Baker in Pilsbry 1946: 178, fig. 84.9; Gittenberger and van Bruggen 2013: 255, fig. 10), *Allopeasclavulinum* (Baker in Pilsbry 1946: fig. 84.6; Marcus and Marcus 1968: fig. 9), *Allopeas* spec. 2 (Gittenberger and van Bruggen 2013: 254–255, fig. 11) and *Allopeas* spec. 3 (Gittenberger and van Bruggen 2013: 255, fig. 12). However the relationships between the penial sheath and the vas deferens seem different from what was illustrated by Marcus and Marcus (1968), the only authors to describe them: according to Marcus and Marcus (1968: fig. 9) the vas deferens runs externally to the penial sheath whereas we found that it runs internally. The proximal penial complex consists of a short bulbous epiphallus and thin walled penial caecum with a branch of the penial retractor on the proximal tip of each (Figs 13, 15, 16). The epiphallus and the penial caecum are so closely juxtaposed as to resemble a usual proximal penis tip with an undivided penial retractor joined to it (Figs 9–12). This arrangement matches that already described in *Allopeasgracile* (Baker 1945: 88; Baker in Pilsbry 1946: 178, fig. 84.10), *Allopeasclavulinum* (Baker 1945: 90; Baker in Pilsbry 1946: 180, fig. 84.6; Marcus and Marcus 1968: fig. 9), *Allopeasmauritianum* (Baker 1945: 90; Baker in Pilsbry 1946: 180) and *Allopeas* spec. 2 (Gittenberger and van Bruggen 2013: 255, fig. 11b). However there seems to be some variation, especially in the structure of the penial caecum between these species: *Allopeasgracile* and *Allopeasmauritianum* have a robust penial caecum, large at the base and progressively tapering towards the tip (for *Allopeasgracile*, see Baker in Pilsbry 1946: fig. 84.10; Gittenberger and van Bruggen 2013; for *Allopeasmauritianum*, see Baker in Pilsbry 1946: fig. 84.3), whereas *Allopeasclavulinum* has a long slender penial caecum almost uniform in diameter, thin along its entire length (Baker in Pilsbry 1946: fig. 84.4; Marcus and Marcus 1968: fig. 9). Based on penial caecum structure, our specimens apparently do not match those assigned to *Allopeasclavulinum* but are more similar to those assigned to *Allopeasmauritianum*. As already rightly observed by Gittenberger and van Bruggen (2013), it is not clear whether these differences are due to individual variation or to phylogenetic divergence. The relationships between these taxa are also uncertain, as is whether these names have been used consistently in the literature. Although they are currently regarded as synonyms (MolluscaBase eds 2024b), their status and relationships are still not clear and may only be defined after the designation of neotypes and study of an appropriate number of populations using an integrative approach with conchological, anatomical and molecular characters. For now we consider this species according to its current concept (e.g., Horsák et al. 2020).

*Allopeasclavulinum* has been found in the tropical greenhouse of MUSE, where it is the commonest and most abundant achatinid species, and in the Biodiversity Garden (Botanical Garden of Padua), where it is rather uncommon. This is the first report from Italy.

#### ﻿ Opeas hannense

Taxon classificationAnimaliaStylommatophoraSubulinidae

(Rang, 1831)

ECB092BC-C5B8-515D-A42A-8B0AF2D7A34A

Helix hannensis Rang, 1831: 41–42, pl. 3, fig. 8. Type locality: Senegal, Cape Verde Peninsula, Hann village (“village de Hann sur la presqu’île du Cap-Verd”). Type material: unknown.

##### Material examined.

Italy • 47 shells and 1 spirit specimen; Trento, Tropical greenhouse of the Science Museum of Trento (MUSE); 46°03'45.16"N, 11°06'50.08"E; 4 Jan. 2019, 10 Feb. 2019, 04 May 2019; D. Barbato, G. Bolzonella leg.; GMC 51196 • 17 shells; same locality; 01 Feb. 2022; D. Barbato, A. Benocci leg.; GMC 51187 • 1 shell; same locality; 02 Feb. 2023; F. Rossi leg.; GMC 57345 • 6 shells; same locality; 09 Feb. 2023; D. Barbato, A. Benocci leg.; GMC 57352.

##### Description.

**Figures 17–20.**
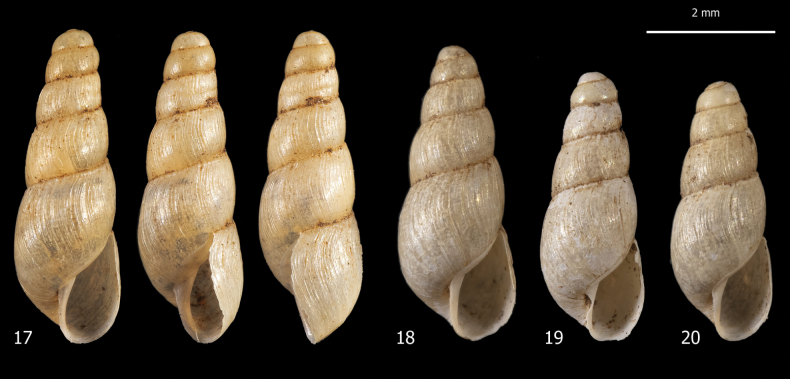
Shells of *Opeashannense* from the tropical greenhouse of the Science Museum of Trento (MUSE), D. Barbato & G. Bolzonella leg. 04 May 2019. Shell material of this species belonged mainly to juveniles; the shells depicted in the figures were the best available, although some are from specimens that were not fully grown.

***Shell*** (Figs 17–20). Dextral, very small in size, very minutely perforate, elongate, very slender, conical, rather robust, pearly off-white, glossy or waxy and sub-transparent when fresh, with 5–6 slightly convex whorls separated by moderately deep sutures. Apex obtuse, rounded, and smooth. Last whorl ~ ½ of shell height and less convex than preceding ones. Aperture small, ~ 1/3 of shell height, obliquely pyriform, slightly prosocline. Peristome interrupted, not thickened, only slightly reflected on columella, sometimes with subtle callous rim on parietum; columella straight; outer margin sinuous in lateral view (approximately inverse S-shaped). Protoconch smooth; teleoconch with weak irregular collabral growth lines. Shell dimensions: SH 4.0–4.8 mm; SD 1.6–1.8 mm; AH 1.6–1.7 mm; AW 0.9–1.0 mm.

***Female distal genitalia*** (Fig. 14). Free oviduct very short and wide. Bursa copulatrix sac-like, oval with short slender duct (as long as bursa copulatrix), initially not flared. Vagina very short and wide (as long as free oviduct).

***Male distal genitalia*** (Fig. 14). Vas deferens almost uniform in diameter (very thin to thin along its entire length), entering penial complex at its proximal end. Penial complex apparently consisting only of penis. Penis short, almost uniformly cylindrical with thin short penial sheath enveloping its distal tract. Penial retractor muscle inserted on proximal end of penis.

***Genital atrium*** (Fig. 14). Rather long.

##### Remarks.

The species was first named *Helixclavulus* by Férussac (1821: 52) based on specimens from Guadeloupe and then *Helixgoodalli* by Miller (1822: 381) on specimens from near Bristol, England. Unfortunately Férussac’s name was not accompanied by a description, a definition, or an indication and so it is not available, whereas Miller’s name, extensively used until the early 20^th^ century (cf. Pilsbry 1906b), turned out to be a junior homonym and was replaced by *Bulimuspumilus* established by Pfeiffer (1840) on specimens from Cuba (cf. Pilsbry 1910). Pilsbry (1906a: 141–142) also discussed the hypothesis of Wollaston (1878: 510) that *Helixgoodalli* was a junior synonym of *Helixhannense* established by Rang (1831) on specimens from the Cape Verde Peninsula, Senegal, observing: “whether this course was well-founded is a question which must remain unsettled until specimens from Rang’s original locality can be compared.” Consequently he never adopted Rang’s name for this species (cf. Pilsbry 1946: 181–182). The synonymy of the two species was reproposed by Groh (1983) based on study of the original descriptions and the literature, and has subsequently been adopted by most recent authors (e.g., von Proschwitz 1994; Cowie 1997, 2000; Chase and Robinson 2001; Bank and Menkhorst 2008; Gerber and Clark 2015; Horsák et al. 2020). Perplexity persists about the real identity of the species described by Rang. Only the dimensions, which are consistent with those of an *Opeas* species, support Groh’s interpretation. Otherwise the situation remains as described by Pilsbry more than a hundred years ago: type material of Rang’s species is unknown; no one has reported or studied material from the type locality, which when Rang visited it, was a small village, today englobed in the city of Dakar (where a green area, the Parc forestier et zoologique de Hann, still survives in Hann); finally Rang’s description and illustration are completely inadequate to establish the identity of the species he treated; his figure depicts a snail with shell having all the whorls quite round, whereas this species has the last whorl almost flat (incidentally Robinson (1999: table 1) considered *Opeashannense* to be absent from Africa).

*Opeashannense* is regarded as native to tropical America (Pilsbry 1946; Kerney and Cameron 1979; Deisler and Abbott 1984; Cowie 1997; Cowie et al. 2008; Brook et al. 2010; Miquel and Herrera 2014) where it is widespread in Central America and the West Indies. On the contrary Robinson (1999: Table 1) regarded it as native to East Asia. It has been introduced into South America, Atlantic islands, East Africa, West Indian Ocean islands, South-east Asia, and Pacific islands. It has also been reported from the mid temperate latitudes of the northern hemisphere where it only occurs in greenhouses and hothouses (see Table 2 for details and references). Since these reports are only based on shell identifications, it is not possible to exclude that some are misidentifications (e.g., Muratov 2010: fig. 28).

**Table 2.** Geographical distribution of *Opeashannense*. Asterisks indicate countries / islands where the species has been recorded only in greenhouses or very disturbed anthropogenic habitats.

Regions	Countries / Islands	References
**North America**	United States*	Dundee (1974), Nekola (2014)
**Central America**	Guatemala, Mexico, Nicaragua, and Panama	Thompson (2011)
**West Indies**	Bahamas, Cuba, Hispaniola, Jamaica, Lesser Antilles (Barbados, Curaçao, Guadeloupe, Martinique, and Saint Martin)	Deisler and Abbott (1984), Chase and Robinson (2001), Rosenberg and Muratov (2006), Espinosa and Ortea (2009), Maceira et al. (2013), Charles (2015), Delannoye et al. (2015), Hovestadt and van Leeuwen (2017), Hovestadt and Neckheim (2020), Espinosa and Robinson (2021)
**South America**	Argentina, Brazil, Ecuador, Suriname, and Uruguay	van Regteren Altena (1975), Simone (2006), Rumi et al. (2010), Virgillito and Miquel (2013), Miquel and Jaime (2018), Breure et al. (2022a)
**Atlantic Ocean**	Bermuda, Capo Verde, Saint Helena, and São Tomé	Crowley and Pain (1977), Groh (1983), Bieler and Slapcinsky (2000), Holyoak et al. (2020), Key et al. (2021), Preece et al. (2022)
**Europe**	Austria*, Czech Republic*, Denmark*, France*, Germany*, Great Britain*, Ireland*, Italy*, Netherlands* and Sweden*	Kerney and Cameron (1979), von Proschwitz (1994), Leiss and Reischütz (1996), Horsák et al. (2004, 2020), Reischütz et al. (2018), Kwitt et al. (2019), Anderson and Rowson (2020), this paper
**Africa**	Mozambique	Muratov (2010)
**Indian Ocean**	Madagascar and Seychelles	Gerlach (2006), Bank and Menkhorst (2008), Emberton et al. (2010)
**South-East Asia**	Singapore	Ho (1995)
**Oceania**	Pacific Islands (American Samoa, Belau/Palau, Cook Islands, Federated States of Micronesia, Fiji, French Polynesia, Galapagos Islands, Guam, Hawaii, Pitcairn Islands, Samoa, Solomon Islands, Tonga, and Vanuatu)	Solem (1989), Cowie (2000, 2001), Brook et al. (2010), Brook (2014), Miquel and Herrera (2014), Cowie et al. (2017)

The genital anatomy of *Opeas* species was investigated by Baker (1945), Baker in Pilsbry (1946), and Gittenberger and van Bruggen (2013). Only three *Opeas* species have been studied: *Opeashannense* (see Baker 1945: 86, as *Opeaspumilum*), *Opeaspyrgula* (Schmacker & Boettger, 1891) (see Baker 1945: 87; Baker in Pilsbry 1946: 183–184, figs 88 2, 3) and an unidentified species from Misali islet, Zanzibar (see Gittenberger and van Bruggen 2013: 251, fig. 9), but only the anatomy of *Opeaspyrgula* is adequately described. The distal genitalia of the only adult specimen that we have been able to study (Fig. 14) agrees with the general scheme described for these species and in particular with the features reported by Baker (1945) and Baker in Pilsbry (1946) for *Opeaspumilum* and *Opeaspyrgula* and with the description of *Opeaspumilum* given by Baker (1945). The major difference between the two species consists in the swelling between the base of the duct of the bursa copulatrix and the proximal vagina: well developed in *Opeaspyrgula* and much less enlarged in *Opeaspumilum*.

Thus, little continues to be known about the genital anatomy of this genus. We need to ascertain whether the proximal complex of the penis is really undivided, to understand the relationships between the vas deferens and the penial sheath and whether the different structure of the female distal genitalia is due to individual variation or to phylogenetic divergence.

*Opeashannense* has only been found in the tropical greenhouse of MUSE, where it is uncommon. This is the first report from Italy.

#### ﻿ Paropeas achatinaceum

Taxon classificationAnimaliaStylommatophoraSubulinidae

(Pfeiffer, 1846)

63A046FF-D7BC-53FD-BB21-A07CD63234F0

Bulimus achatinaceus Pfeiffer, 1846: 82. Type locality: “Java”. Type material: lectotype no. ZMB Moll. 65746, Zoological Museum, Berlin (Naggs 1994: fig. 1).

##### Material examined.

Italy • 173 shells and 21 spirit specimens; Padua, Biodiversity Garden (Botanical Garden of Padua); 45°23'52.59"N, 11°52'50.37"E; 06 Mar. 2019; D. Barbato leg.; GMC 57373.

##### Description.

**Figures 21–24.**
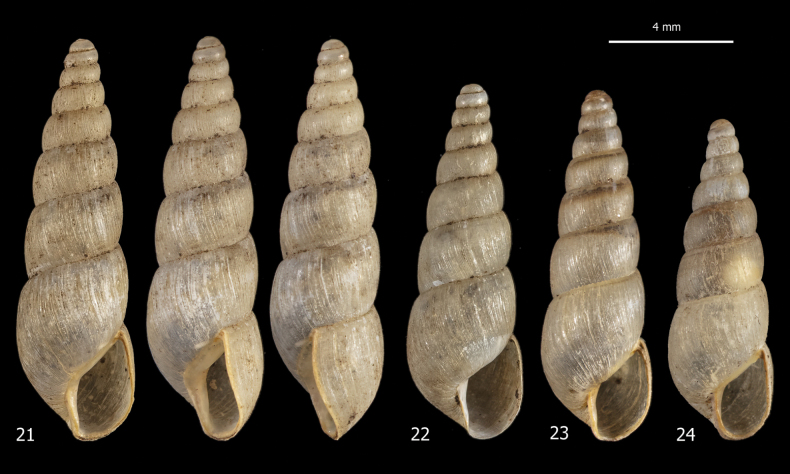
Shells of *Paropeasachatinaceum* from the Biodiversity Garden (Botanical Garden of Padua), D. Barbato leg. 06 Mar. 2019.

***Shell*** (Figs 21–24). Dextral, medium in size, minutely perforate to imperforate, elongate, slender, conical, rather robust, pearly off-white, opaque, with 7–9 slightly convex whorls, separated by rather deep and in places irregularly crenulate sutures. Apex obtuse, rounded, and smooth. Last whorl ~ 1/3 of shell height. Aperture small, ~ 1/4 of shell height, obliquely pyriform, slightly prosocline. Peristome interrupted, slightly thickened along outer margin, slightly reflected on columella, with callous rim on parietum and columella; columella straight; outer margin sinuous in lateral view (approximately inverse S-shaped). Protoconch smooth; teleoconch with evident irregular collabral striae. Shell dimensions: SH 9.4–13.1 mm; SD 2.9–3.8 mm; AH 3.0–3.5 mm; AW 1.8–2.2 mm.

**Figures 25–27.**
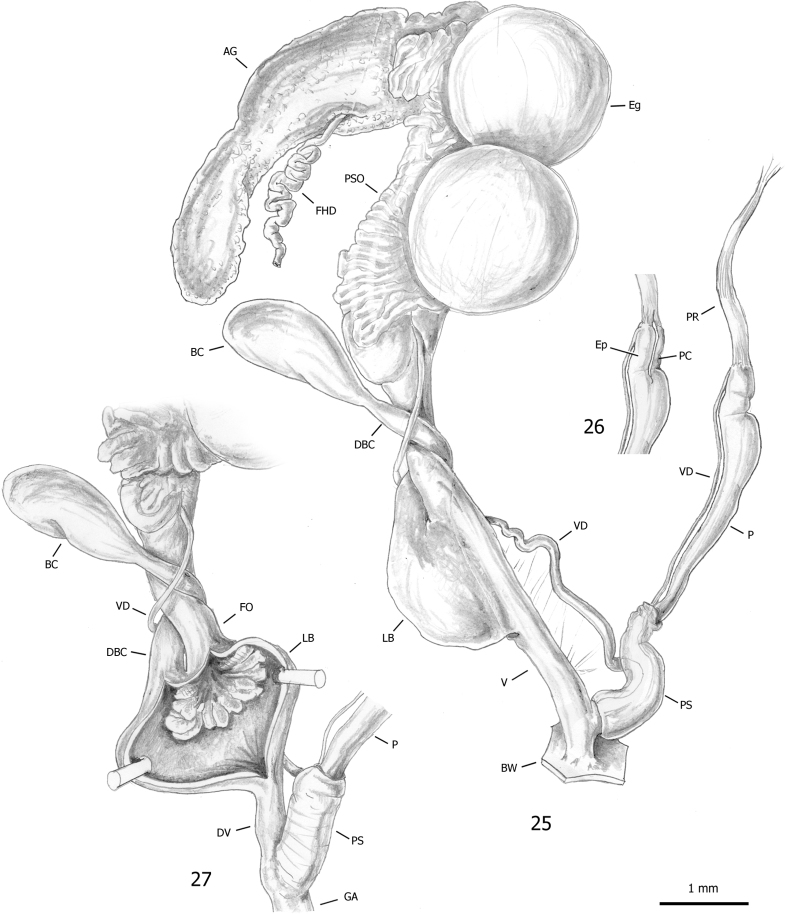
Genital anatomy of *Paropeasachatinaceum* from the Biodiversity Garden (Botanical Garden of Padua), D. Barbato leg. 06 Mar. 2019: **25** genitalia (hermaphrodite gonad excluded) **26** detail of proximal penial complex **27** internal structure of vagina.

***Female distal genitalia*** (Figs 25, 27). Free oviduct long and wide. Bursa copulatrix sac-like, oval with long slender duct (slightly longer than bursa copulatrix), initially slightly flared, and medially convoluted around free oviduct. Vagina long and wide (longer than free oviduct) with huge proximal lateral bulge containing large ligula.

***Male distal genitalia*** (Figs 25–27). Vas deferens of variable diameter (proximal tract narrow, medial tract slightly wider and final tract very narrow), entering penial complex at its proximal end. Penial complex consisting of epiphallus, penial caecum and penis. Epiphallus very short. Penial caecum very short (as long as epiphallus). Penis very long, distinctly divided into proximal and distal parts by difference in calibre; proximal part longer (almost twice distal penis), slender and thin walled; distal penis shorter (half proximal penis), thick, muscular walled and enveloped by penial sheath. Penial retractor muscle bifid, one branch inserted on proximal end of penis, the other branch on tip of penial caecum.

***Genital atrium*** (Figs 25, 27). Short.

##### Remarks.

*Paropeasachatinaceum* is one of the best known subulinids thanks to the excellent anatomical study and the careful taxonomic revision by Naggs (1994). Our anatomical study (Figs 25–27) fully agrees with that of Naggs (1994).

This species is regarded as native to tropical Asia, where it occurs from Nepal and Sri Lanka to South East Asia. Outside this area it is found in Australia and Pacific islands, West Indian Ocean islands, Europe, and the West Asia (see Table 3 for details and references).

**Table 3.** Geographical distribution of *Paropeasachatinaceum*. Asterisks indicate countries / islands where the species has been recorded only in greenhouses or very disturbed anthropogenic habitats; hash symbol denotes one record based on a specimen of unknown origin recovered from sandy detritus collected on a beach.

Regions	Countries / Islands	References
**Europe**	Austria*, Italy*, and Malta**^#^**	Horsák et al. (2020), Cilia et al. (2022), this paper
**West Indian Ocean**	Mascarene and Seychelles	Naggs (1994), Griffiths and Florens (2006)
**West Asia**	Iraq	Hussein et al. (2018)
**South Asia**	Nepal and Sri Lanka	Naggs (1994), Budha et al. (2015)
**South-East Asia**	Borneo, Indonesia, and Singapore	Naggs (1994), Schilthuizen and Rutjes (2001), Tan et al. (2015), Nurinsiyaha et al. (2016), Phung et al. (2017)
**East Asia**	Dongsha Island and Hong Kong	Naggs (1994), Wu et al. (2007)
**Oceania**	Australia and Pacific islands (American Samoa, Cook Islands, Fiji, French Polynesia, Guam, Hawaii, Northern Mariana Islands, Samoa, and Tonga)	Naggs (1994), Cowie (2000, 2001), Shea (2007), Brook et al. (2010), Stanisic et al. (2010), Kerr and Bauman (2013), Brook (2014), Cowie et al. (2017)

The species was found in the Biodiversity Garden (Botanical Garden of Padua), where it forms a well-established population, as in Vienna Zoo, Austria (Horsák et al. 2020). In contrast, the report from Malta is only based on a specimen of unknown origin recovered from sandy detritus collected on a beach (Cilia et al. 2022). This is the first report from Italy and the third from Europe.

#### ﻿ Subulina octona

Taxon classificationAnimaliaStylommatophoraSubulinidae

(Bruguière, 1789)

C3F6AA0A-9993-54F7-8563-FC134A3EC4D8

Bulimus octonus Bruguière, 1789: 325. Type locality: “… dans les îles Antilles. M. de Badier l’a trouvé abondamment à l’île de la Guadeloupe, & j’en ai vu chez M. d’Antic qui lui ont été envoyés de l’île de Saint-Domingue”. Type material: presumed lost (Smith 1992).

##### Material examined.

Italy • 112 shells and 2 spirit specimens; Trento, Tropical greenhouse of the Science Museum of Trento (MUSE); 46°03'45.16"N, 11°06'50.08"E; 04 Jan. 2019; 10 Feb. 2019; 04 May 2019; D. Barbato, G. Bolzonella leg.; GMC 51197 • 29 shells; same locality; 01 Feb. 2022; D. Barbato, A. Benocci leg.; GMC 51188 • 1 shell; same locality; 09 Feb. 2023; D. Barbato, A. Benocci leg.; GMC 57353 • 1 shell; Padua, Biodiversity Garden (Botanical Garden of Padua); 45°23'52.59"N, 11°52'50.37"E; 06 Mar. 2019; D. Barbato leg.; GMC 57374.

##### Description.

**Figures 28–30.**
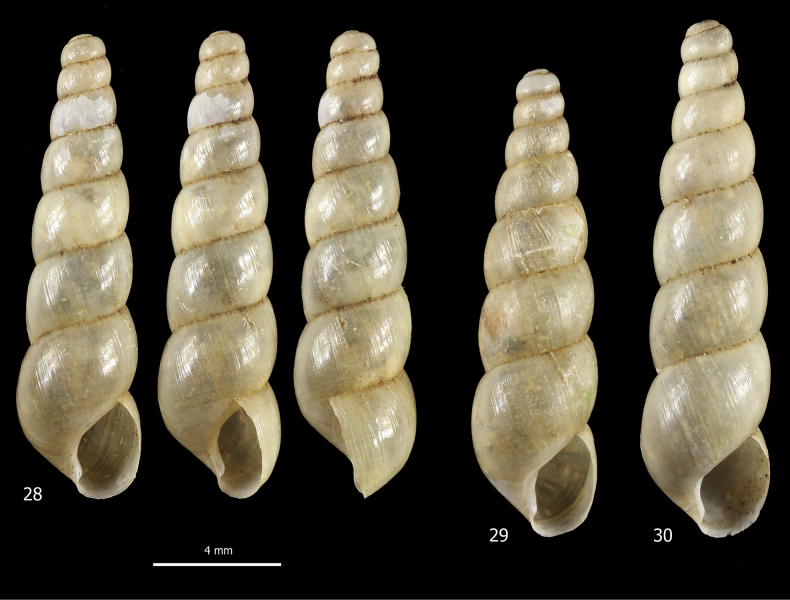
Shells of *Subulinaoctona* from the tropical greenhouse of the Science Museum of Trento (MUSE), D. Barbato & G. Bolzonella leg. 04 Jan. 2019.

***Shell*** (Figs 7, 8, 28–30). Shell dextral, medium in size, imperforate, elongate, slender, conical, rather robust, pearly off-white, glossy or waxy and sub-transparent when fresh, with 7–9 slightly convex whorls, separated by rather deep and in places irregularly crenulate sutures. Apex obtuse, rounded, and smooth; last whorl ~ 1/3 of shell height. Aperture small, ~ 1/5 of shell height, ovate, slightly prosocline. Peristome interrupted, not thickened or reflected, with callous rim on parietum and columella; columella concave and obliquely truncate at base; outer margin straight in lateral view. Protoconch smooth; teleoconch with fine, wrinkled, irregular collabral growth lines. Shell dimensions: SH 12.1–16.0 mm; SD 3.3–4.1 mm; AH 2.5–3.2 mm; AW 1.9–2.4 mm.

**Figures 31, 32.**
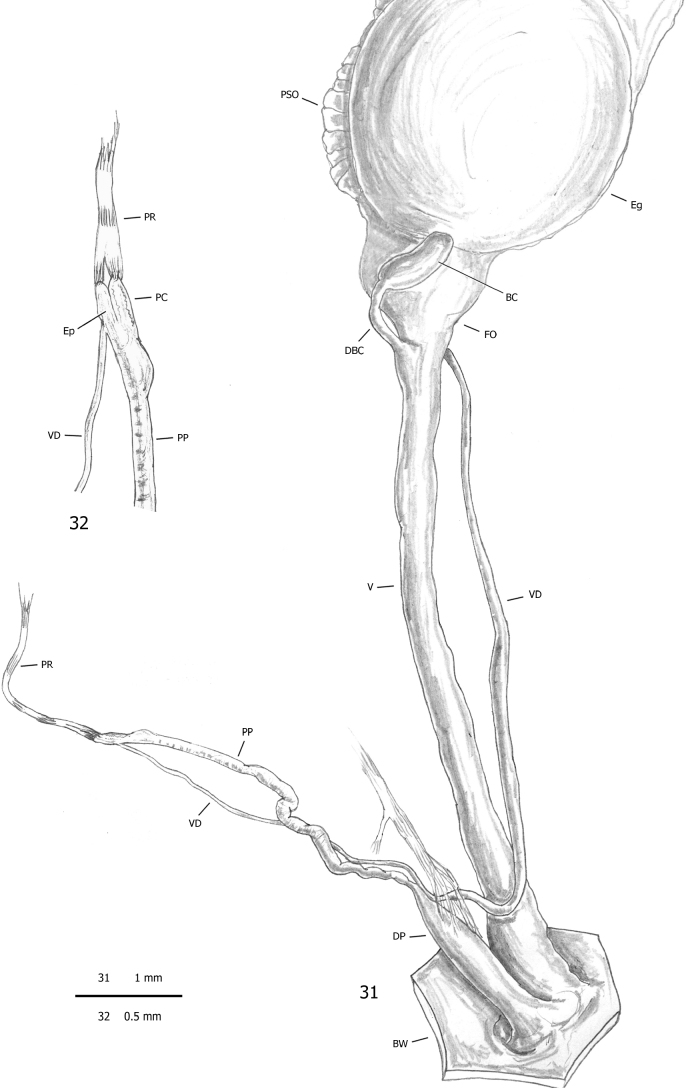
Genital anatomy of *Subulinaoctona* from the tropical greenhouse of the Science Museum of Trento (MUSE), D. Barbato & G. Bolzonella leg. 04 Jan. 2019: **31** genitalia (hermaphrodite gonad excluded) **32** detail of proximal penial complex.

***Female distal genitalia*** (Fig. 31). Free oviduct very short and wide. Bursa copulatrix sac-like, oval with short slender duct (as long as bursa copulatrix), initially not flared. Vagina very long and slender.

***Male distal genitalia*** (Figs 31, 32). Vas deferens of varying diameter (proximal tract narrow, medial tract slightly wider and final tract very narrow), entering penial complex near proximal end. Penial complex consisting of epiphallus, penial caecum and penis. Epiphallus very short. Penial caecum very short (as long as epiphallus). Penis very long, divided distinctly into proximal and distal parts by difference in calibre, without penial sheath; proximal part longer (twice distal penis), slender and thin walled; distal penis shorter (half proximal penis) and thick, muscular walled. Penial retractor muscle bifid, one branch inserted on tip of penial flagellum, the other branch on tip of penial caecum.

***Genital atrium*** (Fig. 31). Very short.

##### Remarks.

*Subulinaoctona* is a well-known travelling snail that occurs worldwide (Robinson 1999) but its native range is uncertain. It was described from Guadeloupe and Hispaniola and has been reported as native to tropical America (Pilsbry 1946), the Caribbean (Deisler and Abbott 1984) and Latin America (Robinson 1999) but this was disputed on the grounds that the other species of the genus occur in Africa (Bieler and Slapcinsky 2000). Gerlach (2006) regarded it as native to the Seychelles, based on a subfossil record from Aldabra reported by Gerlach and van Bruggen (1999). Others suggested that it was originally from tropical America (Griffiths and Florens 2006), south-east Asia (Rumi et al. 2010) or Africa (Breure et al. 2016; Hovestadt and van Leeuwen 2017). Multi-gene phylogenetic analysis of the achatinoid snails by Fontanilla et al. (2017) found that *Subulinaoctona* and *Subulinastriatella* (Rang, 1831) formed a monophyletic group belonging to an unsupported clade including only Old World species, but unfortunately the study included a small selection of subulinine genera, only one of which was from neotropical America.

*Subulinaoctona* is now distributed widely in humid tropical and subtropical lowlands across the world. It occurs in Central America, the West Indies, South America, sub-Saharan Africa, West Indian Ocean islands, South, South-East, and East Asia, New Guinea, Australia, and Pacific islands. In the mid temperate latitudes of the northern hemisphere, it only occurs in greenhouses and hothouses (see Table 4 for details and references).

**Table 4.** Geographical distribution of *Subulinaoctona*. Asterisks indicate countries / islands where the species has been recorded only in greenhouses or very disturbed anthropogenic habitats.

Regions	Countries / Islands	References
**North America**	United States*	Dundee (1974), Nekola (2014)
**Central America**	Belize, Costa Rica, Guatemala, Honduras, Mexico, Nicaragua, Panama, and Salvador	Thompson (2011)
**West Indies**	Bahamas, Cuba, Hispaniola, Jamaica, and Lesser Antilles (Barbados, Curaçao, Guadeloupe, Martinique, Saint Barthélemy, Saint Martin, and Trinidad)	Deisler and Abbott (1984), Chase and Robinson (2001), Rosenberg and Muratov (2006), Espinosa and Ortea (2009), Robinson et al. (2009), Rutherford (2011), Maceira et al. (2013), Charles (2015), Delannoye et al. (2015), Breure et al. (2016), Herrera-Uria (2016), Hovestadt and van Leeuwen (2017), Hovestadt and Neckheim (2020), Espinosa and Robinson (2021)
**South America**	Brazil, Columbia, Ecuador, Peru, Suriname, and Venezuela	Baker (1927), Marcus and Marcus (1968), van Regteren Altena (1975), Simone (2006), Rumi et al. (2010), Breure et al. (2022a)
**Atlantic Ocean**	Bermuda	Bieler and Slapcinsky (2000)
**Europe**	Austria*, Czech Republic*, Denmark*, Great Britain*, Ireland*, Italy*, Netherlands*, and Sweden*	Kerney and Cameron (1979), von Proschwitz (1994, 2016), Juricková (2006), Da Sois (2015), Reischütz et al. (2018), Anderson and Rowson (2020), Horsák et al. (2020), this paper
**Africa**	South Africa, Tanzania, and Zimbabwe	van Bruggen (1981), Rowson (2007), Herbert (2010)
**Indian Ocean**	Aldabra, Madagascar, Maldives, Mascarene, Pemba, and Seychelles	Probst (2001), Gerlach (2006), Griffiths and Florens (2006), Rowson (2007), Emberton and Griffiths (2009), Rowson et al. (2010), Gittenberger and van Bruggen (2013), Gittenberger et al. (2019)
**South Asia**	India and Sri Lanka	Ranawana (2006), Raheem et al. (2014)
**South-East Asia**	Indonesia, Peninsular Malaysia, Sabah in Malaysian Borneo, Singapore, and Vietnam	Schileyko (2011), Tan et al. (2015), Foon et al. (2017), Phung et al. (2017), Nurinsiyaha and Hausdorf (2019)
**East Asia**	Dongsha Islands and Japan*	Minato (1988), Wu et al. (2007)
**Oceania**	Australia, New Guinea, and Pacific islands (American Samoa, Belau/Palau, Cook Islands, Federated States of Micronesia, Fiji, French Polynesia, Galapagos Islands, Guam, Hawaii, Marshall Islands, New Caledonia, Northern Mariana Islands, Pitcairn Islands, Samoa, Solomon Islands, Tonga, and Vanuatu)	Cowie (2000, 2001), Wiktor (2003), Shea (2007), Brook et al. (2010), Rundell (2010), Stanisic et al. (2010), Brodie and Barker (2012), Kerr and Bauman (2013), Brook (2014), Miquel and Herrera (2014), Cowie et al. (2017)

The distal genitalia of MUSE specimens show a general scheme identical to that described by Baker (1927: 3–4, pl. 20, fig. 99), Marcus and Marcus (1968: 189–190) and Araújo and Bessa (1993: 493–495, figs 7–11), but differing in the size of the penial complex from that described by Wiegmann (1894: 214–216, pl. 16, fig. 3; reproduced by Pilsbry 1946: 173, fig. 83h), Schileyko (1999: fig. 662 BC) and Gittenberger and van Bruggen (2013: 250). Instead of a very short penial complex, in agreement with the first description by Baker (1927), MUSE specimens have a long slender penial complex consisting of a short proximal portion (the epiphallus and the penial caecum closely juxtaposed to each other), a long slender medial portion (the proximal penis) and a short swollen distal portion (the distal penis). In particular, based on the male genitalia of a specimen from Dunoon (Guyana) mounted in glycerine jelly and viewed by transmitted light, Baker (1927) described the proximal portion as consisting of a flagellar appendix [the penial caecum] and a very short, thick walled tract [the epiphallus]; the vas deferens entering the penial complex at the proximal end of the epiphallus; the penial retractor joining at the tip of penial caecum; a rather elongate papilla present at the internal opening of the epiphallus into the proximal penis. Baker also described a penial sheath enveloping the distal penis joined by a muscular branch originating from the right lower tentacle retractor; finally he interpreted the structure that Wiegmann (1894) described and figured as the penis as being only the distal penis surrounded by a heavy muscular sheath.

The small size and the very fine structure of the sections of the proximal portion of the penial complex make dissection difficult and differentiation of its components elusive. Our results substantially agree with Baker’s description. The differences are: the vas deferens enters the penial complex near the base and not at the tip of the epiphallus; the penial retractor consists of two branches, one joined to the epiphallus, the other to the penial caecum; the small size of the proximal penis makes the penial papilla impossible to detect by stereomicroscope; a classical penial sheath (such as that of *Allopeas* and *Paropeas*) is absent, although a muscular branch from the right lower tentacle retractor joins the distal penis directly on the penial wall. Unfortunately, the scarcity of material prevented a more careful anatomical examination. Some uncertainties about its real organisation and the meaning of such differences among the various anatomical reports therefore remain.

Multi-gene phylogenetic analysis of the achatinoid snails by Fontanilla et al. (2017) found that the genus, as currently conceived, is polyphyletic because the three *Subulina* species examined did not cluster together. The meaning of variation in penis size in *Subulinaoctona* is also uncertain. Wiegmann (1894) ventured that the extreme reduction of the male distal genitalia was related to “unisexuality” or sequential hermaphroditism (... dass die betreffenden Thiere durch Verkümmerung des männlichen Theils der Genitalien eingeschlechtig geworden sind, oder aber dass die weibliche Geschlechtsreife der männlichen vorausgeht). Others, such as Baker (1927), supposed that the species was a sequential hermaphrodite, but whereas Wiegmann saw it as protogynous, Baker viewed it as protandrous. *Subulinaoctona* is actually a facultatively self-fertilising egg-retaining species showing no evidence of sequential hermaphroditism (Bessa and Araújo 1995; D’Ávila et al. 2018). Thus the reduced penis may be related to loss of biparental reproduction as already supposed by Wiegmann. However this may be because *Subulinaoctona* is a complex of species. More research is needed to address these questions.

*Subulinaoctona* is common in the tropical greenhouse of MUSE, and has been found in the Biodiversity Garden (Botanical Garden of Padua), where only one specimen was collected. This is the first report from Italy.

#### ﻿ Geostilbia aperta

Taxon classificationAnimaliaStylommatophoraSubulinidae

(Swainson, 1840)

11E65CC4-D8CB-53D8-ADEE-277338C7C64A

Macrospira aperta Swainson, 1840: 335, fig. 97e, f. Type locality: no locality given; according to Smith (1892: 269), Saint Vincent, Lesser Antilles, West Indies. Type material: unknown. Note: Swainson attributed the new species to the reverend L. Guilding from St. Vincent, from whom he received the material used for the description. Probably this material was accompanied by a manuscript name which Swainson adopted for denoting the species.
Achatina gundlachi Pfeiffer, 1850: 80. Type locality: Cuba. Type material: unknown.
Geostilbia caledonica Crosse, 1867: 186–187, Pl. 7, fig. 4. Type locality: Nouméa, New Caledonia. Type material: 1 syntype in Crosse collection (MNHN-IM-2000-4720) (Breure et al. 2022b).

##### Material examined.

Italy • 2 shells; Trento, Tropical greenhouse of the Science Museum of Trento (MUSE); 46°03'45.16"N, 11°06'50.08"E; 01 Feb. 2022; D. Barbato, A. Benocci leg.; GMC 51189.

##### Description.

**Figures 33, 34.**
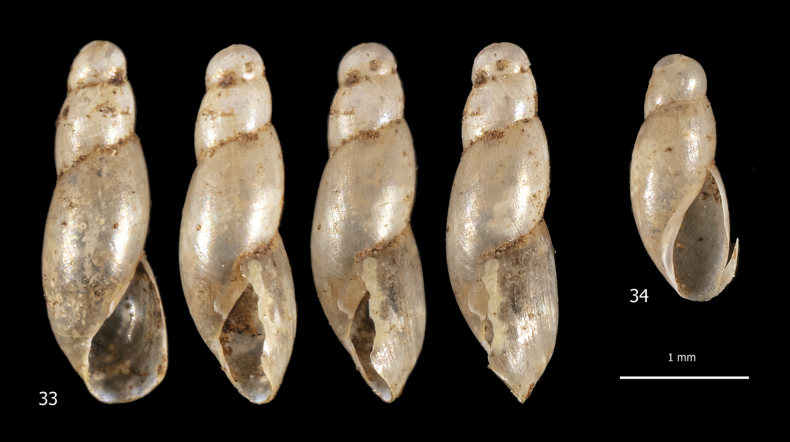
Shells of *Geostilbiaaperta* from the tropical greenhouse of the Science Museum of Trento (MUSE), D. Barbato & A. Benocci leg. 01 Feb. 2022.

***Shell*** (Figs 33, 34). Dextral, very small in size, imperforate, elongate, very slender, cylindro-conical, thin and fragile, pearly off-white, colourless, glossy and transparent when fresh, ~ 4 slightly convex whorls separated by rather deep sutures. Apex obtuse, rounded, and smooth. Last whorl ~ 2/3 of shell height. Aperture small, ~ 1/3 of shell height, ovate to pyriform, basally flared, slightly prosocline. Peristome interrupted, not thickened or reflected, with callous rim on parietum and columella; columella straight or slightly concave; outer margin slightly arched forward in the middle in lateral view. Protoconch smooth; teleoconch with very fine collabral lines and very fine spiral grooves particularly evident on last whorl. Shell dimensions: SH 2.8 mm; SD 1.0 mm; AH 1.1 mm; AW 0.6 mm.

***Body and anatomy*.** Unknown.

##### Remarks.

The early taxonomy of this land snail revolves around three named species: *Macrospiraaperta* Swainson, 1840, *Achatinagundlachi* Pfeiffer, 1850 and *Geostilbiacaledonica* Crosse, 1867.

Commenting on the land mollusc species introduced to Saint Helena, Edgar Smith maintained that *Achatinagundlachi* was a junior synonym of *Macrospiraaperta* based on examination of Guilding’s material from St. Vincent, West Indies, deposited in the British Museum (Smith 1892) and that *Geostilbiacaledonica* was also a junior synonym of *Megaspira* [sic] *aperta* (Smith, 1895).

Pilsbry (1908), in his exhaustive revision of orthurethrous snails included in the second edition of the Manual of Conchology, partly rejected Smith’s conclusions, regarding *Macrospiraaperta* as a species inadequately described and *Geostilbia* as a section of *Cecilioides*. He used *Cecilioidesgundlachi* as the valid name for the species. However many years later, dealing with this group again, he adopted *Cecilioidesaperta* as the valid name (Pilsbry 1946).

No subsequent authors made any significant contribution for a better taxonomic framework of the species. They repeated what Pilsbry (1946) proposed, believing that a species of *Geostilbia*, sometimes considered a subgenus or a synonym of *Cecilioides*, could be found almost everywhere in the world, having spread from the Mesoamerican area (e.g., Robinson 1999). Indeed many consider it native to the West Indies, Neotropics or Caribbean basin (Dundee 1974; Cowie 1997; Robinson 1999; Chase and Robinson 2001; Rosenberg and Muratov 2006; Thompson 2011; Miquel and Herrera 2014; Nurinsiyaha et al. 2016; Nurinsiyaha and Hausdorf 2019). Others report it to be native to southern Europe (Cotton 1954) or North America north of Mexico (Nekola 2014).

A species, sometimes named *Geostilbiaaperta* (or *Cecilioidesaperta*), *Geostilbiacaledonica* (or *Cecilioidescaledonica*) or *Geostilbiagundlachi* (or *Cecilioidesgundlachi*), is currently reported from North, Central and South America, the Caribbean and the Indo-Pacific Region from South-East Asia to Hawaii and Cook Islands (see Table 5 for details and references). It has also been reported from Saint Helena (Smith 1892) based on a misidentification of *Cecilioidesacicula* (Crowley and Pain 1977), and from Barbados, Curaçao and Galapagos, where it was no longer found in recent field surveys (Chase and Robinson 2001; Miquel and Herrera 2014; Hovestadt and van Leeuwen 2017).

**Table 5.** Geographical distribution of *Geostilbiaaperta*. Asterisks indicate countries / islands where the species has been recorded only in greenhouses or very disturbed anthropogenic habitats.

Regions	Countries / Islands	References
**North America**	United States*	Pilsbry (1946), Dundee (1974), Nekola (2014)
**Central America**	Nicaragua	Thompson (2011)
**West Indies**	Cuba, Hispaniola, Jamaica, Lesser Antilles (Guadeloupe, Martinique, Saint Martin, and Saint Vincent)	Smith (1892), Rosenberg and Muratov (2006), Maceira et al. (2013), Charles (2015), Hovestadt and Neckheim (2020), Espinosa and Robinson (2021)
**South America**	Brazil	Simone (2006)
**South-East Asia**	Indonesia, Peninsular Malaysia, and Philippines	Vermeulen and Whitten (1998), Maassen (2001), Groh (2015), Nurinsiyaha et al. (2016), Foon et al. (2017), Nurinsiyaha and Hausdorf (2019)
**Oceania**	Australia, New Guinea, and Pacific Islands (Cook Islands, Guam, Hawaii, and New Caledonia)	Solem (1964), van Benthem Jutting (1964), Cowie (2000, 2001), Shea (2007), Stanisic et al. (2010)

It is difficult to say anything new about this group of species, since there is no anatomical data. We can rely on shell characters, the most interesting of which are the spiral sculpture particularly evident in the last whorl, the non-truncated or slightly truncated columella and the basally flared aperture. Based on a close resemblance to *Cecilioides*, the species of *Geostilbia* have been placed in the family Ferussaciidae but this similarity could also be due to convergence: true *Cecilioides* have no microsculpture on the teleoconch, have a truncated columella and have no basally flared aperture. In their phylogenetic analysis of the achatinoideans Fontanilla et al. (2017) examined a *Cecilioides* species – *Cecilioidesgokweana* (Boettger, 1870) – which may actually be *Geostilbia*, if the material investigated, collected by DG Herbert, matches the description of this species given by him (cf. Herbert 2010: 127 for description and figure). If this is confirmed, *Geostilbia* does not belong to the Ferussaciidae but to a distinct group of Achatinidae. This of course does not resolve the relationships between *Cecilioides* and *Geostilbia*: *Cecilioides* may really belong to the Ferussaciidae or to the same or a different group of Achatinidae, which may include *Geostilbia*.

MolluscaBase lists eight *Geostilbia* species (MolluscaBase eds 2024c), but except for the widespread *Geostilbiaaperta* and the south American *Geostilbiablandiana* Crosse, 1880, all the others occur from Madagascar to Southeast Asia. There is great uncertainty about species-level taxonomy of *Geostilbia*. Some of the species listed by MolluscaBase may prove to be true *Cecilioides* based on the apparent absence of spiral microsculpture, truncated columella and not basally flared aperture: this could be true of *Geostilbiaphilippinica* von Möllendorff, 1890 and *Geostilbiasheilae* Groh, 2015 (see Groh 2015). On the other hand, some species reported as *Cecilioides* by MolluscaBase, such as the East African *Cecilioidescallipeplum* (Connolly, 1923), for which clear spiral sculpture is reported by Verdcourt (1986), van Bruggen and van Goethem (2001), and van Bruggen (2008), or the South African *Cecilioidesgokweana*, for which clear spiral sculpture is described by Herbert (2010), may prove to be true *Geostilbia*.

We assign two shells found in the litter of the tropical greenhouse of MUSE to this species (unfortunately no living specimen was found during our collecting). This is the first report of the species from Europe.

## Supplementary Material

XML Treatment for Allopeas clavulinum

XML Treatment for Opeas hannense

XML Treatment for Paropeas achatinaceum

XML Treatment for Subulina octona

XML Treatment for Geostilbia aperta

## ﻿Additional information

### Author ORCIDs

Giuseppe Manganelli https://orcid.org/0000-0002-8453-280X

Andrea Benocci https://orcid.org/0000-0003-3145-215X

Debora Barbato https://orcid.org/0000-0003-1105-1711

Folco Giusti https://orcid.org/0000-0001-8722-4653

### Data availability

All of the data that support the findings of this study are available in the main text.
